# Peer victimization and social anxiety in adolescence: a comparison between migrant and native students in Italy

**DOI:** 10.3389/fpsyg.2024.1346373

**Published:** 2024-02-29

**Authors:** Daniele Di Tata, Dora Bianchi, Fiorenzo Laghi

**Affiliations:** Department of Developmental and Social Psychology, Sapienza University of Rome, Rome, Italy

**Keywords:** bullying, ethnic discrimination, anxiety, migrants, adolescence

## Abstract

The first aim of this study is to investigate the relationship between bullying victimization and social anxiety in native and migrant adolescents in Italy. Specifically, it was hypothesized that migrant adolescents (in comparison with natives) experience more frequent episodes of bullying victimization, which in turn, may be a risk factor for the development of social anxiety symptoms. The second aim of the study is to explore the relationships from reflected minority categorization to perceived ethnic discrimination at school and social anxiety symptoms, in the subgroup of migrant students. Results showed that the migrant (vs. native) status was predictive of higher scores in social anxiety dimensions (Fear of Negative Evaluation, Social Avoidance and Distress in New Situations, and General Social Avoidance Distress), via the mediating effect of increased peer victimization. Moreover, in the subgroup of migrant participants, an indirect effect of reflected minority categorization on social anxiety was observed, mediated by perceived ethnic discrimination at school. These findings may contribute to the understanding of health inequalities among migrant and native people in the Italian context. Limitations and practical implications of the study were discussed.

## 1 Introduction

Studies investigating the psychophysical health of migrants have given rise to two contrasting theoretical perspectives: the *immigrant advantage theory* and the *migration morbidity hypothesis* (Dimitrova et al., 2017; Tilley et al., 2021). The immigrant advantage theory, often referred to as the *immigrant paradox*, delineates a pattern whereby first-generation migrants, despite having fewer economic and social resources and experiencing greater stress associated with acculturation and migration, report better psychological adjustment than their second-generation counterparts (Garcia Coll and Marks(eds), 2012).

Conversely, the migration morbidity hypothesis, also known as the *acculturation strain theory*, is rooted in the assumption that the migration process results in an increased risk of problematic behaviors among migrant youth (Berry et al., 2006). Specifically, first-generation migrant youth are considered more susceptible to psychological maladjustment than their second-generation migrant or native-born peers, due to the stressors associated with poverty, discrimination, and adaptation to new cultural and linguistic norms (Bhugra, 2004). According to this theory, migrant adolescents are likely to report a higher prevalence of internalizing problems than their counterparts (Katsiaficas et al., 2013), with acculturative stress identified as a crucial predictor of these adverse outcomes (Sirin et al., 2013). Additionally, many first-generation migrant youths are victims of ethnic discrimination, which can increase their risk of developing internalizing problems (Katsiaficas et al., 2013; Maynard et al., 2016). According to the *minority stress model* (Meyer, 1995), individuals belonging to a social minority group face adverse living conditions within their social environment, that place them at greater risk of developing mental health problems. According to Meyer (1995), social minority individuals, including migrants, also face chronic social stress from daily experiences of discrimination and prejudice.

The contrasting findings within the literature emphasize the imperative for a comprehensive investigation into the disparities in mental health between native-born individuals and migrants, analyzing the roles played by individual, societal, and cultural factors. Indeed, recent meta-analytic reviews on this subject (Dimitrova et al., 2016; Shor and Roelfs, 2021; Tilley et al., 2021) have indicated that the psychological adaptation of migrants, be this advantageous or disadvantageous, is not exclusively determined by migratory history. Instead, it is associated with a multifaceted interplay of risk and protective factors that intersect with ethnic heritage, collectively shaping mental health outcomes.

Moreover, adolescence is a crucial stage of human development that requires individuals to navigate many physical, cognitive, emotional, and social changes. These changes expose adolescents to various new social and educational challenges, such as achieving academic success, cultivating close friendships, and gaining peer acceptance. So, during adolescence, individuals find themselves immersed in more extensive and intricate social networks, with peer relationships becoming more and more important in their lives. However, the psychological development of migrant adolescents takes place across various cultures, intersecting numerous acculturation challenges that involve the adoption of cultural and social norms within the receiving community (Jugert and Titzmann, 2020). Simultaneously, they navigate the process of enculturation, wherein they uphold the cultural and social patterns of their family’s origin. Considering that these acculturative tasks coexist with normative developmental challenges, it is crucial to comprehensively understand how the migratory background affects the formation of positive peer relationships in adolescence, ultimately contributing to the risk of mental health issues during this stage of life (Suárez-Orozco et al., 2018; Jugert and Titzmann, 2020).

Bullying victimization refers to the deliberate and repetitive infliction of harm upon an individual by one or more peers, who typically possess some form of power advantage (Farrington, 1993; Olweus, 1993). This definition encompasses a wide range of aggressive acts, which can be classified into four types: (1) verbal aggression; (2) physical attack; (3) relational or social aggression; and (4) cyberbullying (Olweus, 1993; Smith, 2014; Menesini and Salmivalli, 2017). Different individual and social factors contribute to determining the precise power imbalance that characterizes the relationship between a bully and a victim (Menesini and Salmivalli, 2017). For this reason, not all adolescents face the same risk of bullying victimization. Many studies have examined the factors influencing the risk of victimization during adolescence, yielding substantial evidence regarding the predictive roles played by various individual and interpersonal factors (Card et al., 2008; Card and Hodges, 2008; Troop-Gordon, 2017). Specifically, some authors have observed that migrant and ethnic minority youth tend to be more frequently harassed by children and adolescents (Graham and Juvonen, 2002; Alivernini et al., 2019; Pistella et al., 2020). According to the *social misfits theory* (Wright et al., 1986), ethnic minority students may be more frequent targets of bullying behavior due to their deviation from the norms of the majority group, particularly when they belong to a culture that is very different from the dominant one.

Previous research has demonstrated the existence of a relationship between bullying victimization and psychological maladjustment, specifically concerning internalizing problems such as depression and anxiety (Hawker and Boulton, 2000; Reijntjes et al., 2010; Wu et al., 2015; Silberg et al., 2016; Arseneault, 2017). Within this literature, particular attention has been given to social anxiety. Social anxiety entails a pervasive fear of and avoidance of social situations, driven by the anticipation of negative evaluations by others (Stein and Stein, 2008). These symptoms can manifest in specific situations or across social contexts, causing significant psychological distress that may have broader implications for an individual’s overall functioning (Katzelnick and Greist, 2001).

Peer relationships involving negative evaluations are likely to exert the most significant influence on the development and persistence of social anxiety during adolescence (Wong and Rapee, 2016). Regarding potential mechanisms through which bullying victimization may contribute to subsequent internalizing issues (e.g., social anxiety), developmental theories suggest that negative beliefs and self-evaluations often induced by this type of social experience may lead to enduring problems related to self-esteem and negative emotions (Wang, 2011). In line with this, recent meta-analytic studies have provided empirical evidence for the predictive role played by peer victimization in the development of internalizing symptoms (Moore et al., 2017; Liao et al., 2022).

While there is clear evidence indicating that peer victimization can lead to the development of internalizing symptoms such as anxiety and depression, there is also a growing body of research highlighting a bidirectional relationship (Christina et al., 2021; Liao et al., 2022). Indeed, according to symptom-driven models, children and adolescents with internalizing symptoms may exhibit behaviors or emotional responses that make them more susceptible to peer victimization (Carthy et al., 2010; Forbes et al., 2019).

The present study aimed at investigating bullying victimization experiences and social anxiety among native and migrant adolescents in Italy. Specifically, the research pursued two main objectives: (1) analyzing the indirect effect of migrant (vs. native) status on the presence of social anxiety symptoms, mediated by bullying victimization; and (2) investigating the associations between reflected minority categorization, perceived ethnic discrimination at school and the presence of social anxiety symptoms in the subgroup of migrant students.

Based on previous research (Alivernini et al., 2019; Pistella et al., 2020; Stevens et al., 2020), it was hypothesized that there would be more reports of bullying victimization experiences among migrant students, compared to natives. Consequently, higher victimization was expected to be associated with more severe social anxiety symptoms. According to the minority stress model (Meyer, 1995, 2003; Clark et al., 1999) and social misfits theory (Wright et al., 1986), it was further hypothesized that migrant students who feel perceived by others as members of an ethnic minority group, would be more vulnerable to developing internalizing symptoms such as social anxiety, due to the heightened risk of experiencing discrimination.

## 2 Materials and methods

### 2.1 Participants and procedures

The sample comprised 527 secondary school students (45% girls; 18% migrants), aged 16 to 21 years (*M*_*age*_ = 17.47, SD = 1.01), enrolled in two schools in the province of Rome. Concerning the ethnic composition of the migrants’ subsample, 64% of participants indicated their origin as Eastern Europe, 16% as Western Europe, 9% as Africa, 7% as Latin America, 3% as North America, and 1% each as Asia and Australia, respectively. Following approval from the school administrators, informative letters and consent forms were distributed to students and parents. Subsequently, self-report questionnaires were administered during regular school hours. Students who provided verbal assent and had parental authorization completed paper-and-pencil questionnaires over a duration of approximately 30 min. In each classroom, two research assistants were present to explain the activity, provide instruction, and address any participant concerns. Confidentiality of the results and the voluntary nature of participation were ensured. The study was approved by the Ethical Committee of the Department of Developmental and Social Psychology of Sapienza University of Rome.

### 2.2 Measures

#### 2.2.1 Bullying victimization

Following the definition of victimization experiences that adolescents may encounter in the school context, the Florence Victimization and Bullying Scales (Palladino et al., 2016, 2020) were administered to investigate the frequency with which participants had experienced or engaged in various forms of physical, verbal, and indirect aggression over the prior 2 months. The Florence Victimization and Bullying Scales are comprised of 22 items rated on 5-point Likert scale ranging from 1 (never) to 5 (several times a week). Among these, 11 items ask about victimization (e.g., “I have been physically attacked”), with 4 items specifically addressing ethnic-based bullying (e.g., “I have been excluded due to my ethnicity”). The remaining 11 items assess bullying behaviors (e.g., “I made fun of someone”), with 4 items focused on ethnic-based bullying (e.g., “I spread rumors about someone because of their ethnicity”). The scale has been validated in the Italian context, showing good psychometric properties (Palladino et al., 2016). In the present study, only the victimization subscale was used, with the exclusion of items related to ethnic victimization. The decision to exclude these specific items was driven by the aim to avoid overlap in research objectives and mitigate potential bias. This ensures that the study’s focus on traditional victimization does not inadvertently lead to the assumption that students with a migratory background experience bullying more frequently than their native counterparts due to racial motives. The instrument demonstrated good statistical reliability, with a Cronbach’s alpha score of 0.80.

#### 2.2.2 Social anxiety

The Social Anxiety Scale for Adolescents (SAS-A; La Greca and Lopez, 1998, Italian version by Bianchi et al., 2020) was employed to assess non-clinical levels of social anxiety among participants, considering both the presence and frequency of symptoms in their social interactions. The scale is comprised of 18 items rated on a 5-point Likert scale ranging from 1 (not at all true) to 5 (always true). It includes three subscales: Fear of Negative Evaluation (FNE: 8 items; e.g., “I am afraid they are making fun of me”), Social Avoidance and Distress Specific to New Situations or Unfamiliar Peers (SAD-New: 6 items; e.g., “I become nervous when I talk to people I know little”), and Social Avoidance and Distress that is Experienced More Generally in Social Interactions (SAD-General: 4 items; e.g., “It is difficult for me to ask others to do something together”). The scale has previously been validated in the Italian context, demonstrating good psychometric properties (Bianchi et al., 2020). In the current study, all three subscales demonstrated good statistical reliability, with Cronbach’s alpha scores of 0.83 for SAD-General, 0.90 for SAD-New, 0.92 for FNE, and 0.95 for overall social anxiety.

#### 2.2.3 Perceived ethnic discrimination

The short version of the Perceived Ethnic Discrimination Questionnaire-Community Version (PEDQ-CV; Brondolo et al., 2006, Italian translation by Andrighetto et al., 2013) was used to examine migrant adolescents’ perceived ethnic discrimination at school. The questionnaire consists of 16 items (e.g., “Due to my ethnic background. classmates make me feel different because of my appearance”) rated on a 7-point Likert scale ranging from 1 (never) to 7 (very often). Items assess four aspects of perceived discrimination: exclusion, stigmatization/devaluation, institutional discrimination, and threat/aggression. The present study utilized a composite score of perceived ethnic discrimination, calculated as the mean of all items. The scale demonstrated good statistical reliability, with a Cronbach’s alpha score of 0.82. The instrument was administered exclusively to migrant participants.

#### 2.2.4 Reflected minority categorization

To assess reflected minority categorization, an item utilized in prior investigations (Ferrari et al., 2022a,b) was employed. Using a 4-point Likert scale ranging from 1 (never) to 4 (often), participants were asked to evaluate the frequency with which they had experienced the following situation: “When a stranger meets me, they assume I am a foreigner rather than Italian.” Specifically, this item was used to measure the extent to which participants felt perceived by others as a member of a minority ethnic group.

#### 2.2.5 Sociodemographic variables

Participants provided information about their gender, age, and ethnic background. Regarding the latter, participants were asked to indicate their own and their parents’ countries of birth. Based on established criteria (Suárez-Orozco et al., 2018; Gönültaş and Mulvey, 2021), individuals with at least one foreign-born parent were operationally categorized as migrant youth. Furthermore, a distinction was made between first-generation migrants (i.e., born abroad from foreign-born parents) and second-generation migrants (i.e., born in the host country from foreign-born parents). Conversely, adolescents were classified as non-migrant (i.e., native) youth if they and both parents were born in Italy.

### 2.3 Data analysis

Statistical analyses were conducted using Jamovi version 2.3 (The Jamovi Project, 2022). Initially, descriptive statistics and bivariate correlations were computed for all study variables. To preliminarily assess differences in victimization experiences between native and migrant students, a general linear mixed model (GLMM) was estimated, using school class as the clustering variable. The model included migrant status and gender as factors, with age as a covariate. Fixed effects and the interaction effect between migrant background and gender were analyzed, while controlling for the fixed effect of age and accounting for the random effect of intra-school class variation (see Supplementary Tables 2.1, 2.2, for the results).

Thereafter, a multivariate mediation model was estimated on the whole sample, to investigate the indirect pathways linking migrant (vs. native) background with the three dimensions of social anxiety (i.e., Fear of Negative Evaluation, Social Avoidance and Distress in New Situations, General Social Avoidance and Distress), mediated by experienced victimization. For these purposes, migrant background was dummy coded (0 = native; 1 = first- or second-generation migrant), in the light of the GLMM results. Participants’ gender and age were included as covariates.

Finally, a simple mediation model was tested exclusively in the subsample of migrant participants, to analyze the mediating role played by perceived ethnic discrimination in the relationship between reflected minority categorization and social anxiety in migrant adolescents. Social anxiety was entered as total score in this second model, in order to maximize statistical power due to the reduced sample size. Gender, age, and migrant background (first vs. second generation) effects were controlled as covariates.

For both models, bootstrapping with 5,000 samples (Preacher and Kelley, 2011) was conducted to estimate the indirect effects and 95% confidence intervals (CIs).

## 3 Results

### 3.1 General linear mixed model

A general linear mixed model (GLMM) was estimated to examine the effect of migratory background on school victimization experiences while accounting for gender differences and age. School course acted as a random factor. The results indicated a significant effect of migrant status on victimization, *F*(2, 518) = 5.57, *p* < 0.01. Specifically, Tukey’s *post hoc* comparison showed that both first- and second-generation migrants reported more frequent victimization experiences at school relative to native students (Table 1). However, no significant differences in victimization emerged between first- and second-generation migrants. Moreover, no statistically significant differences were observed with respect to gender, *F*(21, 493) = 0.08, *p* = 0.78, or age, *F*(1, 129) = 0.02, *p* = 0.89. The interaction effect between gender and migrant background was not statistically significant, *F*(2, 515) = 0.38, *p* = 0.68. The random factor accounted for 3% of the variance in victimization.

**TABLE 1:** Descriptive statistics of the victimization scores for each group.

	Natives M(DS)	First-generation migrants M(DS)	Second-generation migrants M(DS)
Victimization	1.34 (0.47)^b^	1.60 (0.85)^a^	1.51 (0.57)^a^

Different letters indicate significant mean differences among groups: a > b.

### 3.2 Multivariate mediation model

**FIGURE 1:**
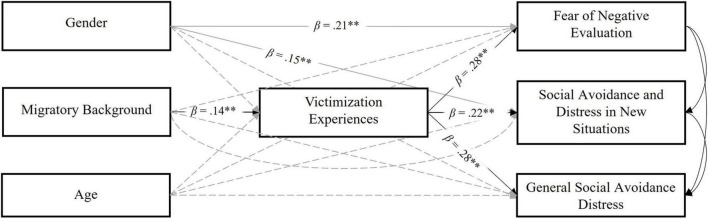
Path diagram for the multivariate mediation model. Control paths are shown in gray. Gender (0 = male, 1 = female); Migratory Background (0 = native, 1 = migrant). **p < 0.01.

A multivariate mediation model was conducted to test the indirect effect of migrant status on social anxiety dimensions through the frequency of victimization experiences. Gender and age were included as covariates to control for their effects. Based on the preliminary results of the GLMM, which indicated no significant differences in victimization between first- and second-generation migrants, these groups were aggregated into a single group for the multivariate mediation analysis. Figure 1 presents the path coefficient estimates.

The results demonstrated a significant effect of migrant status on school victimization. Specifically, migrant students reported more frequent victimization experiences relative to their native counterparts. Additionally, a significant and positive effect of victimization frequency on social anxiety symptoms was observed across all three dimensions (i.e., Fear of Negative Evaluation, Social Avoidance and Distress in New Situations, General Social Avoidance and Distress). The analysis also revealed a significant effect of gender on Fear of Negative Evaluation and Social Avoidance and Distress in New Situations, with higher scores observed among girls.

The analysis of indirect effects showed that victimization mediated the relationships between migrant status and Fear of Negative Evaluation (*B* = 0.12, *SE*_*B*_ = 0.04 [0.04, 0.20], *p* < 0.01), Social Avoidance and Distress in New Situations (*B* = 0.09, *SE*_*B*_ = 0.03 [0.03, 0.16], *p* < 0.01), and General Social Avoidance and Distress (*B* = 0.11, *SE*_*B*_ = 0.04 [0.04, 0.18], *p* < 0.01). Thus, migrant adolescents reported higher levels of victimization, constituting a risk factor for various aspects of social anxiety. The model explained a significant 12% of the variance in Fear of Negative Evaluation, 8% of the variance in Social Avoidance and Distress in New Situations, and 9% of the variation in General Social Avoidance and Distress.

Considering the bidirectional relationship between victimization and social anxiety documented in previous research, we have examined an alternative model to assess the indirect effect of migratory background on experienced victimization mediated by symptoms of social anxiety. The mediational analysis did not reveal significant indirect effects of migratory background on victimization, mediated by Fear of Negative Evaluation, (*B* = 0.01, *SE*_*B*_ = 0.01 [−0.02, 0.04], *p* = 0.67), Social Avoidance and Distress in New Situations, (*B* = −0.01, *SE*_*B*_ = 0.01 [−0.04, 0.01], *p* = 0.32), and General Social Avoidance and Distress, (*B* = 0.03, *SE*_*B*_ = 0.02 [0.00, 0.07], *p* = 0.14), respectively.

### 3.3 Mediation model

**FIGURE 2:**
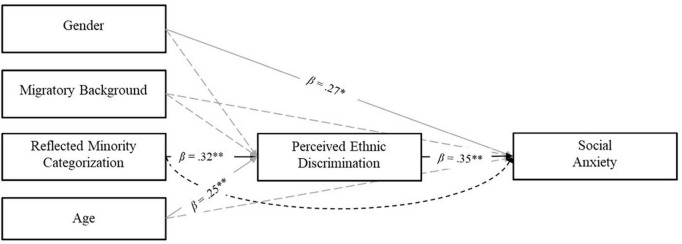
Path diagram for the mediation model. Controls paths are shown in gray. Significant paths of interest are represented by solid lines. Dashed lines represent the non-significant paths of interest. Gender (0 = male, 1 = female); Migratory Background (0 = native, 1 = migrant). *p < 0.05, **p < 0.01.

Only in the subgroup of migrant participants (*n* = 95), a simple mediation model was estimated to analyze the mediating role of perceived ethnic discrimination in the relationship between migratory background, reflected minority categorization, and social anxiety among first- and second-generation migrant adolescents. Gender, age, and migrant background were included as covariates to control for their effects. Figure 2 reports the path coefficient estimates.

The results revealed a significant indirect effect of reflected minority categorization on social anxiety, mediated by perceived ethnic discrimination (*B* = 2.23, *SE*_*B*_ = 0.98 [0.33, 4.14], *p* < 0.05). Adolescents who perceived that others categorized them as members of an ethnic minority group reported higher levels of perceived ethnic discrimination. In turn, perceived ethnic discrimination resulted a positive and significant predictor of social anxiety. Regarding the control variables, significant and positive effects of gender (with higher scores observed among girls) and age on perceived ethnic discrimination were observed. The model explained a significant 16% of the variance in social anxiety.

## 4 Discussion

The present study investigated victimization experiences and social anxiety among native and migrant adolescents in Italy. More specifically, the study examined the indirect impact of migrant status on social anxiety symptoms mediated by peer victimization, and explored the associations between reflected minority categorization, perceived ethnic discrimination within the school context, and social anxiety symptoms among migrant students.

Respect to differences in victimization between native and migrant students, the preliminary analysis indicated that, in the 2 months preceding the research, both first- and second-generation migrant students experienced more frequent school victimization relative to their native counterparts. However, no statistically significant differences were observed between first- and second-generation migrants with respect to this factor. These findings align with prior studies (Strohmeier et al., 2011; Alivernini et al., 2019; Pistella et al., 2020) showing an elevated risk of victimization among ethnic minority students. The dynamics of bully–victim interactions, characterized by an imbalance of power (Olweus, 1993), likely reflect broader societal interactions between majority and minority groups in society (McKenney et al., 2006; Vervoort et al., 2010), with minority groups facing social and economic disadvantages marked by exclusion and discrimination across various facets of life. This marginalized social status, combined with prejudiced attitudes held by members of the native community toward ethnic minorities (Griffiths and Nesdale, 2006; Váradi, 2014), may foster an environment in which migrants are more prone to victimization.

Additionally, the multivariate mediation model showed significant indirect effects of migrant status on all three dimensions of social anxiety: Fear of Negative Evaluation, Social Avoidance and Distress in New Situations, and General Social Avoidance Distress. Hence, migrant adolescents in the study experienced more frequent episodes of victimization, which, in turn, may have constituted a risk factor for their development of social anxiety symptoms. This pattern aligns with the minority stress model (Meyer, 1995, 2003), suggesting that some individuals experience chronic stress from stigmatization and victimization due to their minority background. This form of social stress constitutes a significant risk factor for psychological health, operating through processes influenced by both distal and proximal stressors that fuel shame, fear, and negative social expectations linked to one’s identity (Meyer, 2003). In these terms, it could be hypothesized that migrant adolescents who have experienced victimization may internalize relational attacks, thereby developing a negative self-evaluation and heightened psychological distress, both during and in anticipation of peer aggression, as suggested by prior research (e.g., Grills and Ollendick, 2002; Storch et al., 2005). Furthermore, as a coping mechanism, adolescents may selectively avoid social interactions in which they perceive a risk of aggression (Storch et al., 2005).

Finally, among migrant participants, an indirect effect of reflected minority categorization on social anxiety was observed, mediated by perceived ethnic discrimination at school. This finding supports the minority stress model (Meyer, 1995, 2003), indicating a direct association between stigmatization and social anxiety symptoms among ethnic minority adolescents. Moreover, the result emphasizes that the risk of discrimination and its psychological consequences is not solely determined by an individual’s migratory background, but also by the extent to which they are perceived as deviating from the majority culture. As suggested by Strohmeier et al. (2011), some migrants may face social barriers and communication challenges due to perceived physical and cultural differences or language difficulties, leading to potential rejection and stigmatization within their peer group. This aligns with both *social identity theory* (Tajfel et al., 1979) and social misfit theory (Wright et al., 1986), which suggest that individuals who deviate from the dominant group and are perceived as outgroup members are more likely to face discrimination experiences, that negatively affect psychological functioning (Doğan and Strohmeier, 2020).

It is important to note the limitations of the present study. First, the data referred to diverse and small migrant groups comprised of individuals of varying ethnic backgrounds, making it challenging to differentiate between groups. Second, although a distinction was made between first-generation migrants and second-generation migrants, information regarding first-generation migrant youths’ length of residence in the receiving country was lacking. Third, the absence of longitudinal data prevented the description of causal relationships. Future research employing longitudinal designs is needed to gain a deeper understanding of how migrant background might influence peer victimization and its associated negative outcomes over time. Fourth, the reliance on self-report measures introduced the potential for inaccuracies and desirability bias in respondents’ answers. Future research should explore migrant victimization risk using multi-informant measurement methodologies to provide a more comprehensive view of inter-ethnic relationships in the school context. Finally, the utilization of convenience sampling raises concerns about selection bias, thereby limiting the generalizability of the results.

Despite the abovementioned limitations, the present findings may inform the development of prevention or intervention programs to foster positive inter-ethnic relationships among adolescents of diverse ethnic backgrounds. Indeed, adolescents are significantly influenced by their social interactions, which could affect mental health outcomes. Recognizing the influential role of peer relationships in acculturative and social adolescent development is essential for policymakers, educators, and healthcare professionals in order to foster inclusive school environments. Building upon the specific findings of this study, school interventions should acknowledge that the risk of discrimination is tied not only to migratory background but also to perceptions of deviation from the majority culture. Emphasizing perceived similarity over atypicality is crucial for promoting positive relationships between native and migrant students, presenting a preventive approach to reduce victimization and social anxiety in ethnic minority youth.

## Data availability statement

The raw data supporting the conclusions of this article will be made available by the authors, without undue reservation.

## Ethics statement

The studies involving humans were approved by the Ethical Committee of the Department of Developmental and Social Psychology at Sapienza University of Rome. The studies were conducted in accordance with the local legislation and institutional requirements. Written informed consent for participation in this study was provided by the participants’ legal guardians/next of kin.

## Author contributions

DDT: Conceptualization, Data curation, Formal Analysis, Funding acquisition, Investigation, Methodology, Visualization, Writing – original draft, Writing – review & editing. DB: Conceptualization, Supervision, Writing – review & editing. FL: Conceptualization, Funding acquisition, Supervision, Writing – review & editing.
