# Supplementary material for: Peer victimization and social anxiety in adolescence: a comparison between migrant and native students in Italy
Source: Front Psychol. 2024 Feb 29;15:1346373. doi: 10.3389/fpsyg.2024.1346373 (PMC10937586; doi:10.3389/fpsyg.2024.1346373)
Supplement: Supplementary file 1 [file Data_Sheet_1.docx]

Supplementary Material

Peer Victimization and Social Anxiety in Adolescence: A Comparison between Migrant and Native Students in Italy

**Daniele Di Tata, Dora Bianchi, Fiorenzo Laghi***

**Correspondence:** Fiorenzo Laghi: fiorenzo.laghi@uniroma1.it

# Supplementary Figures and Tables

| **Table 2.1**. *Descriptive statistics and bivariate correlations among study variables in native and migrant students* | | | | | | | |
| --- | --- | --- | --- | --- | --- | --- | --- |
|  | 1. | 2. | 3. | 4. | 5. | 6. | 7. |
| 1. Gender | - |  |  |  |  |  |  |
| 2. Age | -.01 | - |  |  |  |  |  |
| 3. Migrant status | .01 | .09* | - |  |  |  |  |
| 4. Victimization | .02 | .02 | .15** | - |  |  |  |
| 5. SAD - New | .16** | .02 | .09* | .23** | - |  |  |
| 6. SAD - General | .03 | .00 | .10* | .29** | .76** | - |  |
| 7. FNE | .22** | -.04 | .02 | .28** | .76** | .70** | - |
| *M (SD)* | - | 17.46  (1.01) | - | 1.38  (.51) | 2.77  (1.14) | 2.22  (1.06) | 2.75  (1.16) |
| *Scale range* | 0-1 | 16-21 | 0-1 | 1-5 | 1-5 | 1-5 | 1-5 |

*Note*. SAD – New = Social Avoidance and Distress in New Situations; SAD – General = General Social Avoidance and Distress; FNE = Fear of Negative Evaluation; Gender (0 = male, 1 = female); Migrant status (0 = native, 1 = migrant).

* *p* < .05, ** *p* < .01

| **Table 2.2.** *Descriptive statistics and bivariate correlations among study variables only in migrant students (N=95)* | | | | | | |
| --- | --- | --- | --- | --- | --- | --- |
|  | 1. | 2. | 3. | 4. | 5. | 6. |
| 1. Gender | - |  |  |  |  |  |
| 2. Age | -.26* | - |  |  |  |  |
| 3. Migrant generation | -.05 | .04 | - |  |  |  |
| 4. Reflected Minority Categorization | -.09 | .09 | .01 | - |  |  |
| 5. Perceived Ethnic Discrimination | -.15 | .19 | -.06 | .33** | - |  |
| 6. Social Anxiety | .22* | -.04 | .13 | .11 | .31* | - |
| *M* (*SD*) | - | 17.66  (1.06) | - | 1.93  (.99) | 1.48  (.55) | 2.78  (1.10) |
| *Scale range* | 0-1 | 16-21 | 0-1 | 1-5 | 1-5 | 1-5 |

*Note*. SAD – New = Social Avoidance and Distress in New Situations; SAD – General = General Social Avoidance and Distress; FNE = Fear of Negative Evaluation; Gender (0 = male, 1 = female); Migrant status (0 = native, 1 = migrant).

* *p* < .05, ** *p* < .01
